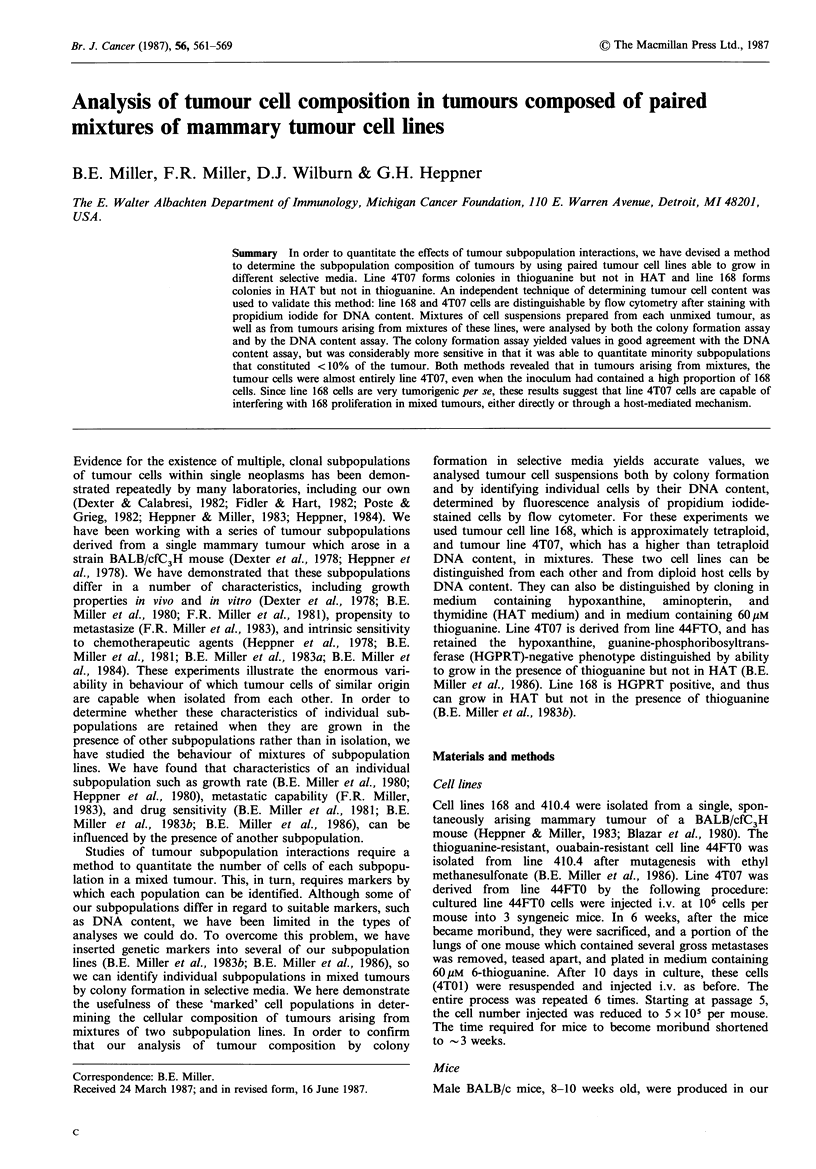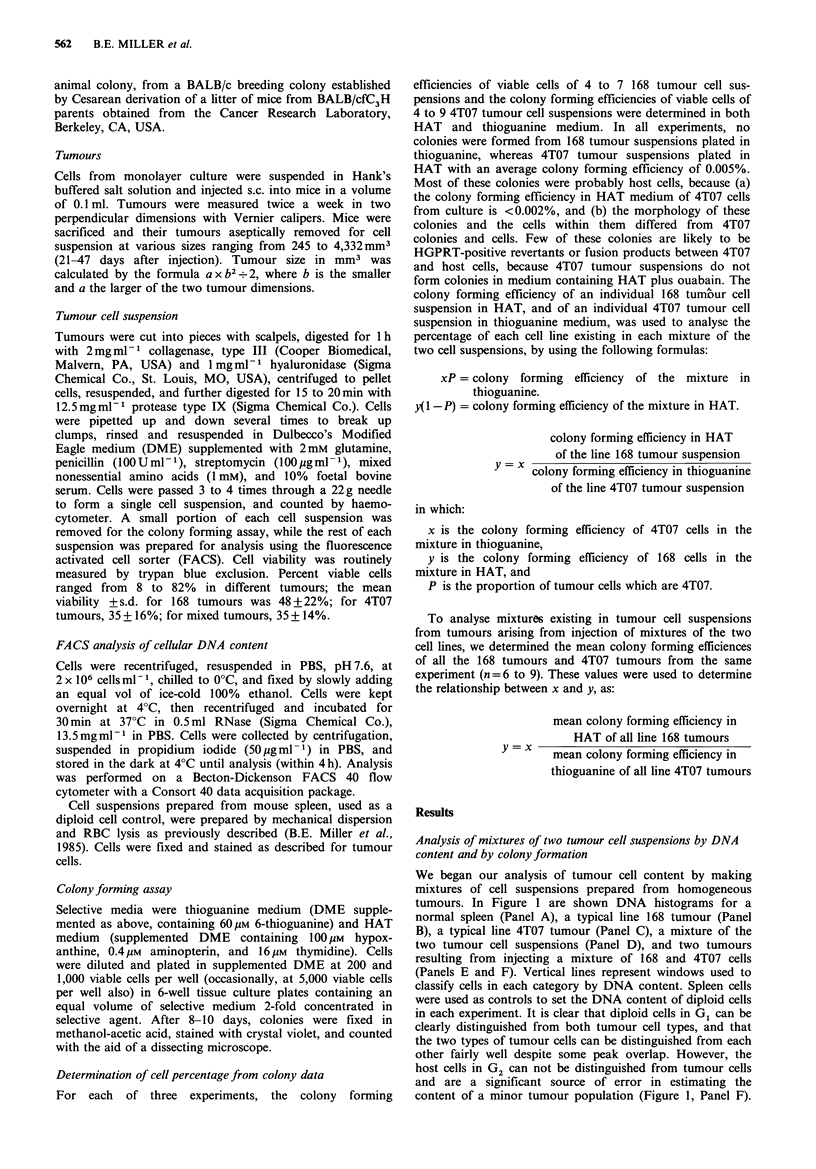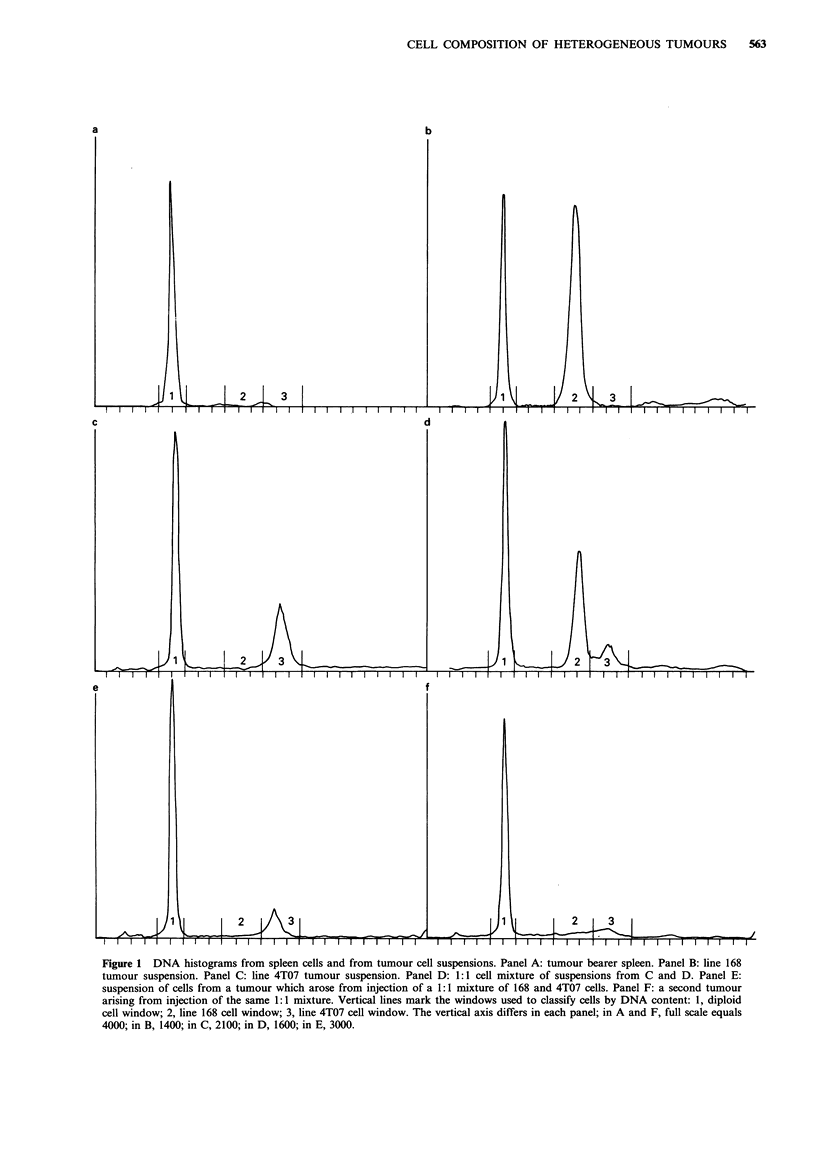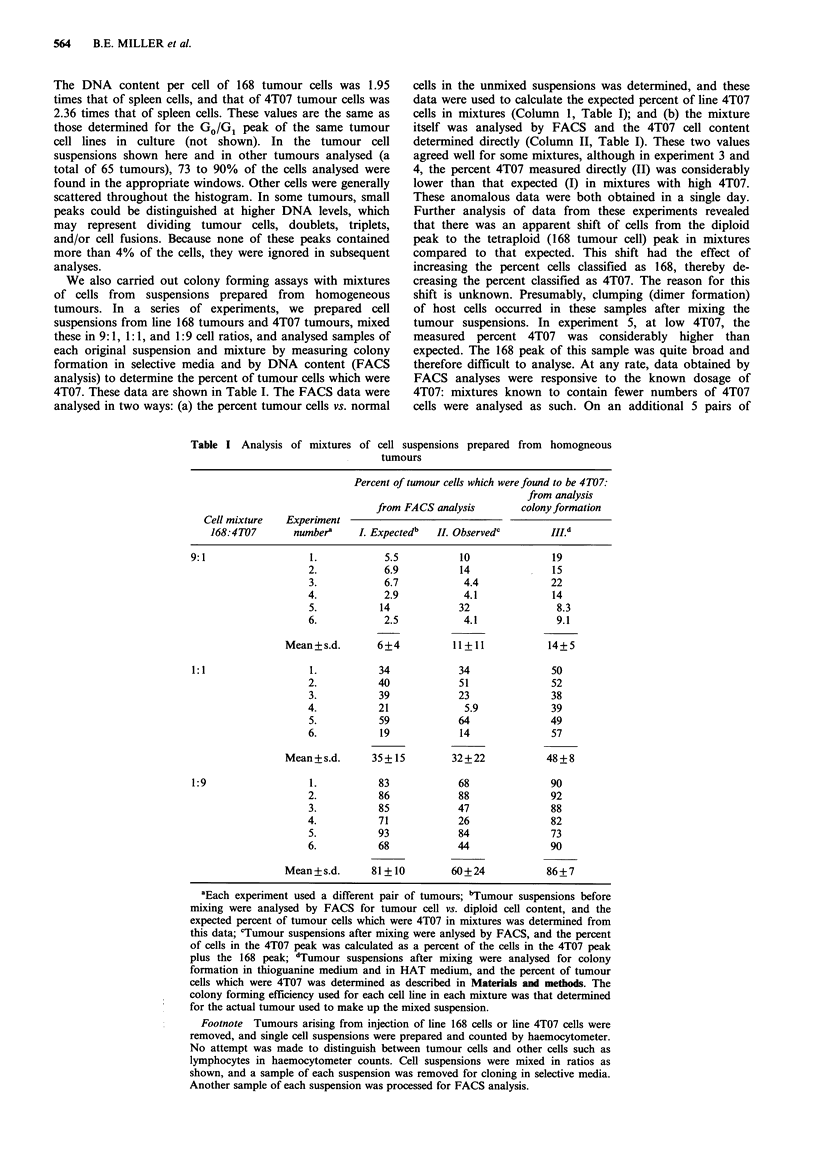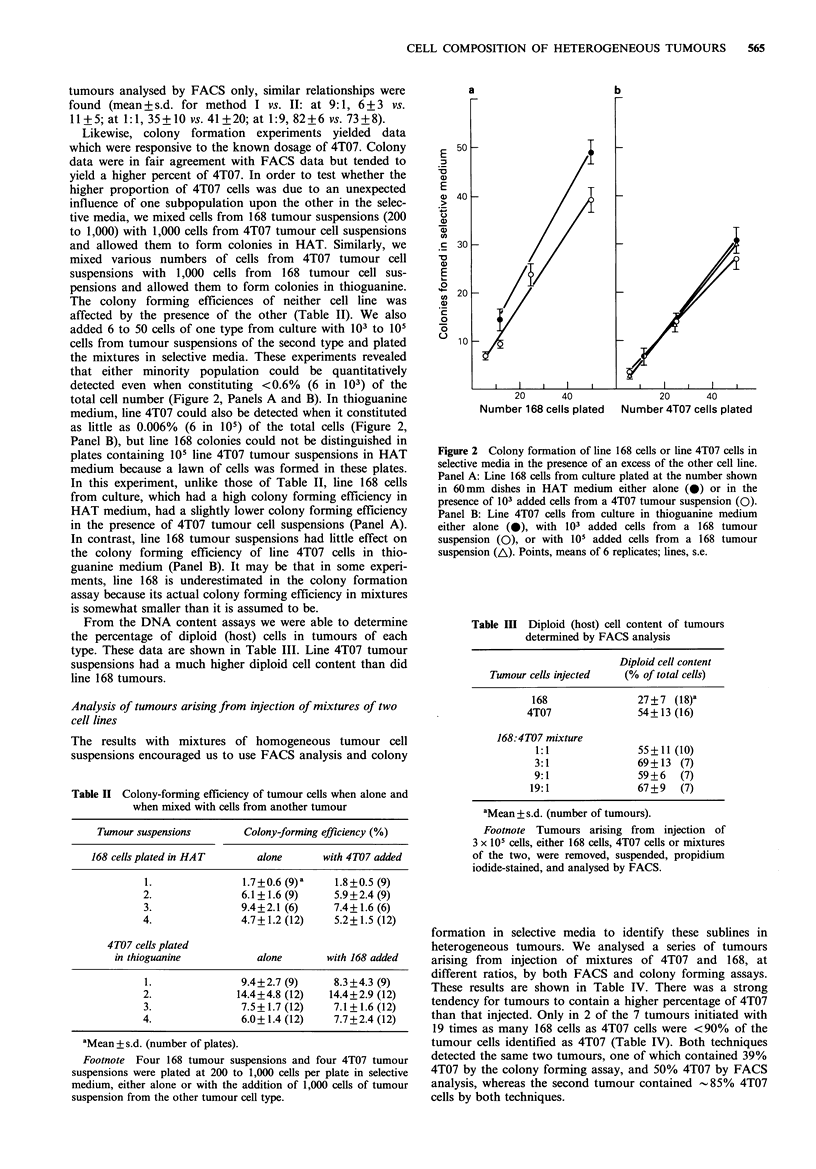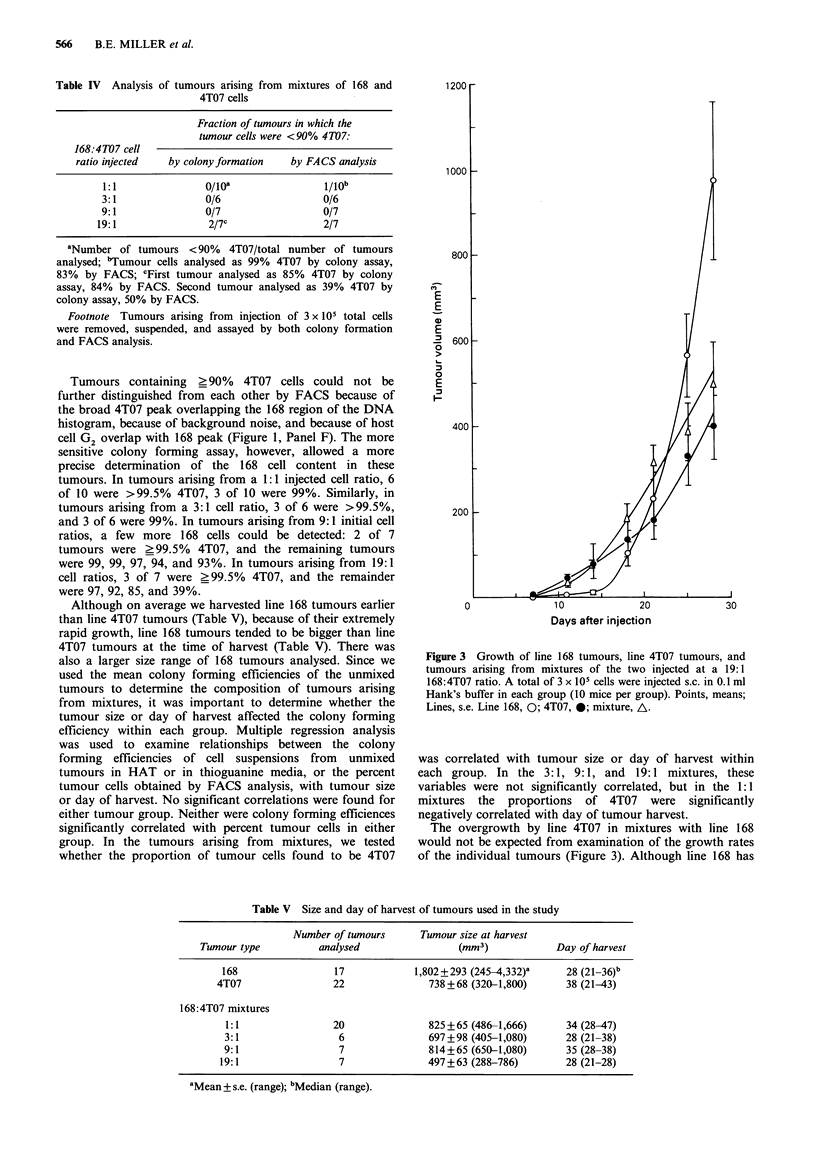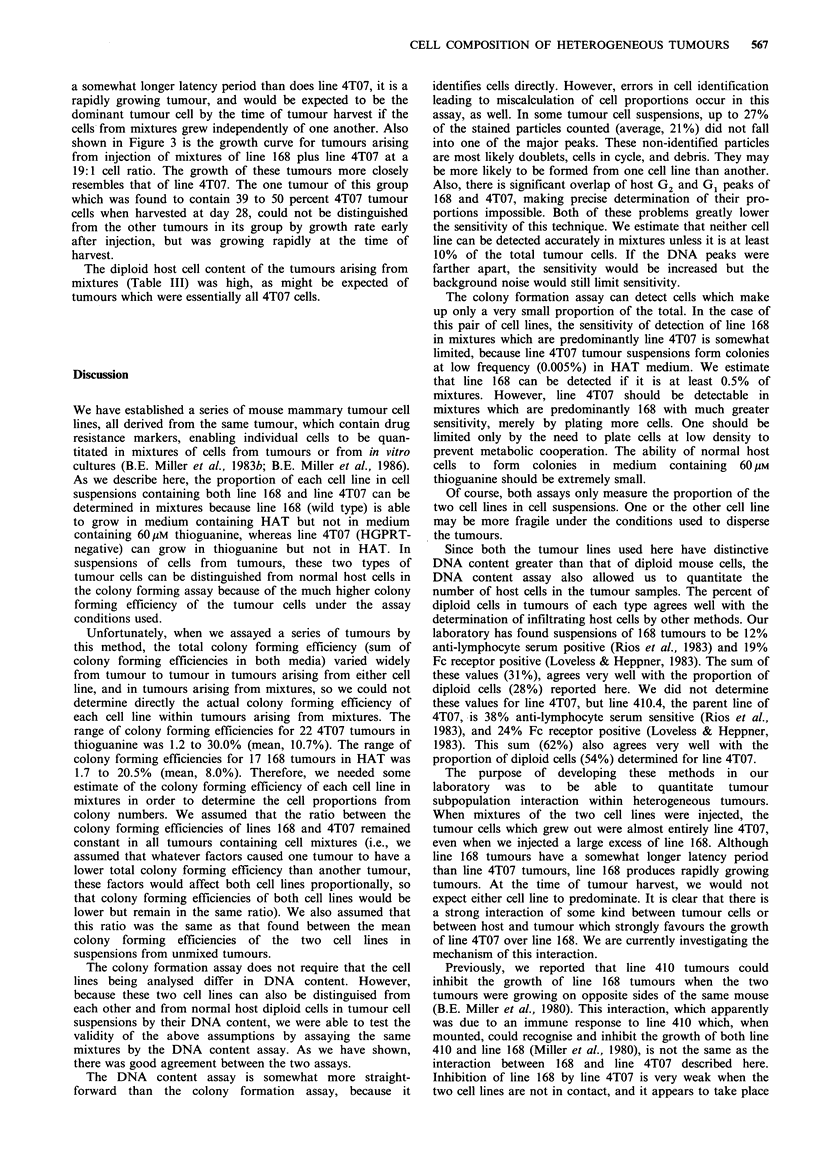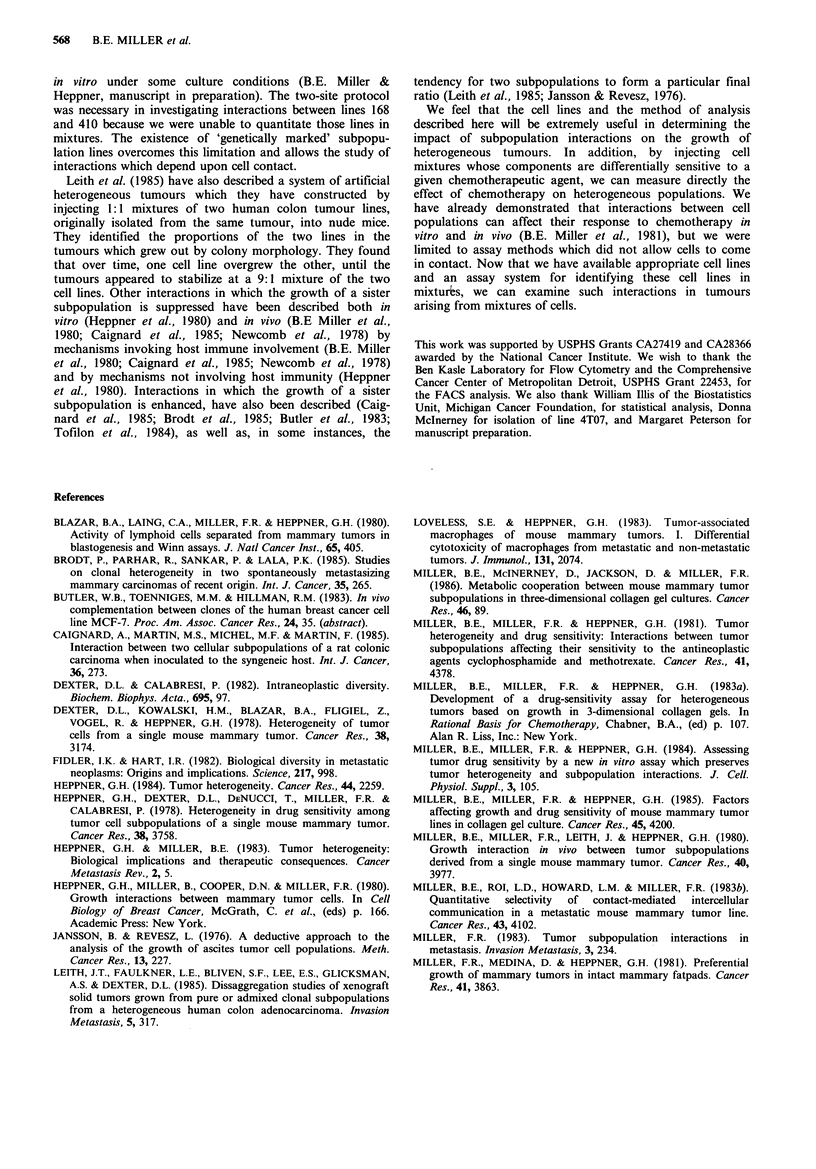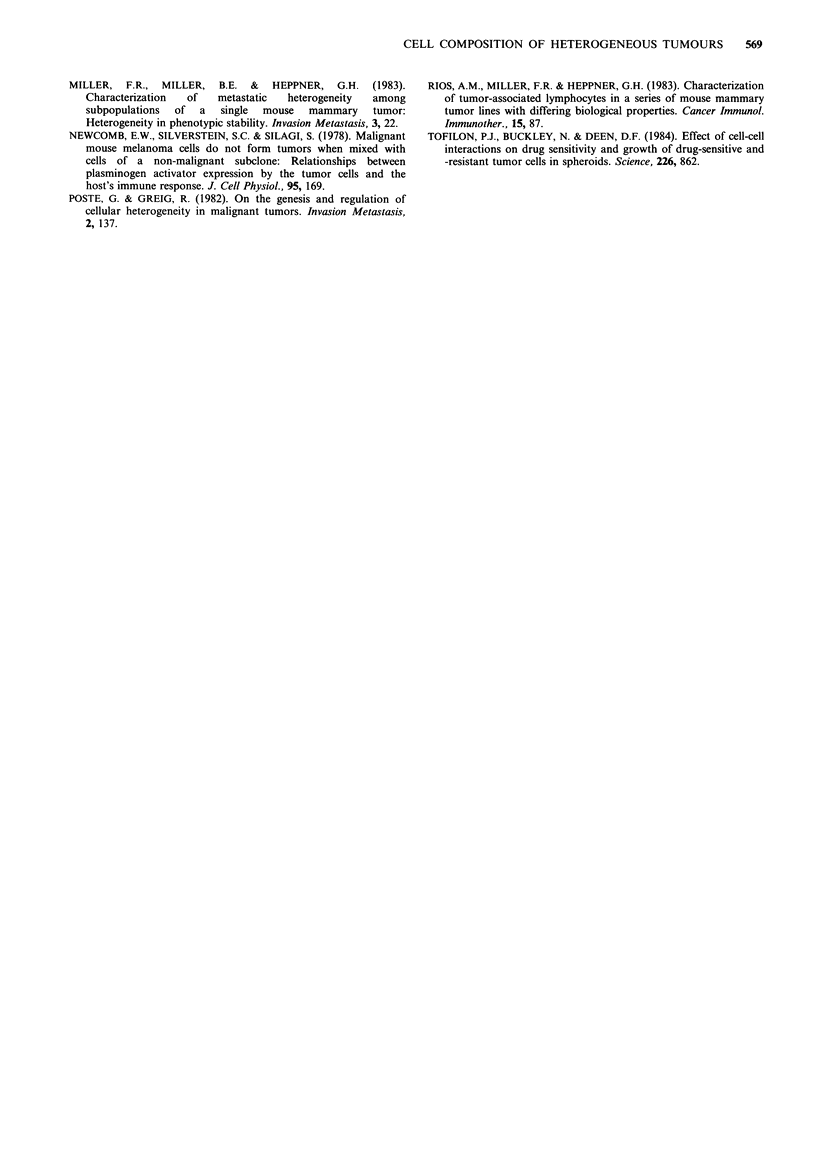# Analysis of tumour cell composition in tumours composed of paired mixtures of mammary tumour cell lines.

**DOI:** 10.1038/bjc.1987.242

**Published:** 1987-11

**Authors:** B. E. Miller, F. R. Miller, D. J. Wilburn, G. H. Heppner

**Affiliations:** E. Walter Albachten Department of Immunology, Michigan Cancer Foundation, Detroit 48201.

## Abstract

In order to quantitate the effects of tumour subpopulation interactions, we have devised a method to determine the subpopulation composition of tumours by using paired tumour cell lines able to grow in different selective media. Line 4T07 forms colonies in thioguanine but not in HAT and line 168 forms colonies in HAT but not in thioguanine. An independent technique of determining tumour cell content was used to validate this method: line 168 and 4T07 cells are distinguishable by flow cytometry after staining with propidium iodide for DNA content. Mixtures of cell suspensions prepared from each unmixed tumour, as well as from tumours arising from mixtures of these lines, were analysed by both the colony formation assay and by the DNA content assay. The colony formation assay yielded values in good agreement with the DNA content assay, but was considerably more sensitive in that it was able to quantitate minority subpopulations that constituted less than 10% of the tumour. Both methods revealed that in tumours arising from mixtures, the tumour cells were almost entirely line 4T07, even when the inoculum had contained a high proportion of 168 cells. Since line 168 cells are very tumorigenic per se, these results suggest that line 4T07 cells are capable of interfering with 168 proliferation in mixed tumours, either directly or through a host-mediated mechanism.


*Br. J. Cancer* (1987), **56**, 561–569 © The Macmillan Press Ltd., 1987

### Analysis of tumour cell composition in tumours composed of paired mixtures of mammary tumour cell lines

B.E. Miller, F.R. Miller, D.J. Wilburn & G.H. Heppner

*The E. Walter Albachten Department of Immunology, Michigan Cancer Foundation, 110 E. Warren Avenue, Detroit, MI 48201, USA.*

**Summary** In order to quantitate the effects of tumour subpopulation interactions, we have devised a method to determine the subpopulation composition of tumours by using paired tumour cell lines able to grow in different selective media. Line 4T07 forms colonies in thioguanine but not in HAT and line 168 forms colonies in HAT but not in thioguanine. An independent technique of determining tumour cell content was used to validate this method: line 168 and 4T07 cells are distinguishable by flow cytometry after staining with propidium iodide for DNA content. Mixtures of cell suspensions prepared from each unmixed tumour, as well as from tumours arising from mixtures of these lines, were analysed by both the colony formation assay and by the DNA content assay. The colony formation assay yielded values in good agreement with the DNA content assay, but was considerably more sensitive in that it was able to quantitate minority subpopulations that constituted <10% of the tumour. Both methods revealed that in tumours arising from mixtures, the tumour cells were almost entirely line 4T07, even when the inoculum had contained a high proportion of 168 cells. Since line 168 cells are very tumorigenic *per se*, these results suggest that line 4T07 cells are capable of interfering with 168 proliferation in mixed tumours, either directly or through a host-mediated mechanism.

Evidence for the existence of multiple, clonal subpopulations of tumour cells within single neoplasms has been demonstrated repeatedly by many laboratories, including our own (Dexter & Calabresi, 1982; Fidler & Hart, 1982; Poste & Grieg, 1982; Heppner & Miller, 1983; Heppner, 1984). We have been working with a series of tumour subpopulations derived from a single mammary tumour which arose in a strain BALB/cf$C_3$H mouse (Dexter *et al.*, 1978; Heppner *et al.*, 1978). We have demonstrated that these subpopulations differ in a number of characteristics, including growth properties *in vivo* and *in vitro* (Dexter *et al.*, 1978; B.E. Miller *et al.*, 1980; F.R. Miller *et al.*, 1981), propensity to metastasize (F.R. Miller *et al.*, 1983), and intrinsic sensitivity to chemotherapeutic agents (Heppner *et al.*, 1978; B.E. Miller *et al.*, 1981; B.E. Miller *et al.*, 1983*a*; B.E. Miller *et al.*, 1984). These experiments illustrate the enormous variability in behaviour of which tumour cells of similar origin are capable when isolated from each other. In order to determine whether these characteristics of individual subpopulations are retained when they are grown in the presence of other subpopulations rather than in isolation, we have studied the behaviour of mixtures of subpopulation lines. We have found that characteristics of an individual subpopulation such as growth rate (B.E. Miller *et al.*, 1980; Heppner *et al.*, 1980), metastatic capability (F.R. Miller, 1983), and drug sensitivity (B.E. Miller *et al.*, 1981; B.E. Miller *et al.*, 1983*b*; B.E. Miller *et al.*, 1986), can be influenced by the presence of another subpopulation.

Studies of tumour subpopulation interactions require a method to quantitate the number of cells of each subpopulation in a mixed tumour. This, in turn, requires markers by which each population can be identified. Although some of our subpopulations differ in regard to suitable markers, such as DNA content, we have been limited in the types of analyses we could do. To overcome this problem, we have inserted genetic markers into several of our subpopulation lines (B.E. Miller *et al.*, 1983*b*; B.E. Miller *et al.*, 1986), so we can identify individual subpopulations in mixed tumours by colony formation in selective media. We here demonstrate the usefulness of these 'marked' cell populations in determining the cellular composition of tumours arising from mixtures of two subpopulation lines. In order to confirm that our analysis of tumour composition by colony formation in selective media yields accurate values, we analysed tumour cell suspensions both by colony formation and by identifying individual cells by their DNA content, determined by fluorescence analysis of propidium iodide-stained cells by flow cytometer. For these experiments we used tumour cell line 168, which is approximately tetraploid, and tumour line 4T07, which has a higher than tetraploid DNA content, in mixtures. These two cell lines can be distinguished from each other and from diploid host cells by DNA content. They can also be distinguished by cloning in medium containing hypoxanthine, aminopterin, and thymidine (HAT medium) and in medium containing 60 $\mu$M thioguanine. Line 4T07 is derived from line 44FTO, and has retained the hypoxanthine, guanine-phosphoribosyltransferase (HGPRT)-negative phenotype distinguished by ability to grow in the presence of thioguanine but not in HAT (B.E. Miller *et al.*, 1986). Line 168 is HGPRT positive, and thus can grow in HAT but not in the presence of thioguanine (B.E. Miller *et al.*, 1983*b*).

### Materials and methods

### *Cell lines*

Cell lines 168 and 410.4 were isolated from a single, spontaneously arising mammary tumour of a BALB/cf$C_3$H mouse (Heppner & Miller, 1983; Blazar *et al.*, 1980). The thioguanine-resistant, ouabain-resistant cell line 44FT0 was isolated from line 410.4 after mutagenesis with ethyl methanesulfonate (B.E. Miller *et al.*, 1986). Line 4T07 was derived from line 44FT0 by the following procedure: cultured line 44FT0 cells were injected i.v. at $10^6$ cells per mouse into 3 syngeneic mice. In 6 weeks, after the mice became moribund, they were sacrificed, and a portion of the lungs of one mouse which contained several gross metastases was removed, teased apart, and plated in medium containing 60 $\mu$M 6-thioguanine. After 10 days in culture, these cells (4T01) were resuspended and injected i.v. as before. The entire process was repeated 6 times. Starting at passage 5, the cell number injected was reduced to $5 \times 10^5$ per mouse. The time required for mice to become moribund shortened to ~3 weeks.

### *Mice*

Male BALB/c mice, 8–10 weeks old, were produced in our

Correspondence: B.E. Miller.
Received 24 March 1987; and in revised form, 16 June 1987.

animal colony, from a BALB/c breeding colony established by Cesarean derivation of a litter of mice from BALB/cf$C_3$H parents obtained from the Cancer Research Laboratory, Berkeley, CA, USA.

*Tumours*

Cells from monolayer culture were suspended in Hank's buffered salt solution and injected s.c. into mice in a volume of 0.1 ml. Tumours were measured twice a week in two perpendicular dimensions with Vernier calipers. Mice were sacrificed and their tumours aseptically removed for cell suspension at various sizes ranging from 245 to 4,332 $mm^3$ (21–47 days after injection). Tumour size in $mm^3$ was calculated by the formula $a \times b^2 \div 2$, where $b$ is the smaller and $a$ the larger of the two tumour dimensions.

*Tumour cell suspension*

Tumours were cut into pieces with scalpels, digested for 1 h with 2 mg $ml^{-1}$ collagenase, type III (Cooper Biomedical, Malvern, PA, USA) and 1 mg $ml^{-1}$ hyaluronidase (Sigma Chemical Co., St. Louis, MO, USA), centrifuged to pellet cells, resuspended, and further digested for 15 to 20 min with 12.5 mg $ml^{-1}$ protease type IX (Sigma Chemical Co.). Cells were pipetted up and down several times to break up clumps, rinsed and resuspended in Dulbecco's Modified Eagle medium (DME) supplemented with 2 mM glutamine, penicillin (100 U $ml^{-1}$), streptomycin (100 $\mu g$ $ml^{-1}$), mixed nonessential amino acids (1 mM), and 10% foetal bovine serum. Cells were passed 3 to 4 times through a 22 g needle to form a single cell suspension, and counted by haemocytometer. A small portion of each cell suspension was removed for the colony forming assay, while the rest of each suspension was prepared for analysis using the fluorescence activated cell sorter (FACS). Cell viability was routinely measured by trypan blue exclusion. Percent viable cells ranged from 8 to 82% in different tumours; the mean viability $\pm$s.d. for 168 tumours was $48 \pm 22\%$; for 4T07 tumours, $35 \pm 16\%$; for mixed tumours, $35 \pm 14\%$.

*FACS analysis of cellular DNA content*

Cells were recentrifuged, resuspended in PBS, pH 7.6, at $2 \times 10^6$ cells $ml^{-1}$, chilled to 0°C, and fixed by slowly adding an equal vol of ice-cold 100% ethanol. Cells were kept overnight at 4°C, then recentrifuged and incubated for 30 min at 37°C in 0.5 ml RNase (Sigma Chemical Co.), 13.5 mg $ml^{-1}$ in PBS. Cells were collected by centrifugation, suspended in propidium iodide (50 $\mu g$ $ml^{-1}$) in PBS, and stored in the dark at 4°C until analysis (within 4 h). Analysis was performed on a Becton-Dickenson FACS 40 flow cytometer with a Consort 40 data acquisition package.

Cell suspensions prepared from mouse spleen, used as a diploid cell control, were prepared by mechanical dispersion and RBC lysis as previously described (B.E. Miller *et al.*, 1985). Cells were fixed and stained as described for tumour cells.

*Colony forming assay*

Selective media were thioguanine medium (DME supplemented as above, containing 60 $\mu$M 6-thioguanine) and HAT medium (supplemented DME containing 100 $\mu$M hypoxanthine, 0.4 $\mu$M aminopterin, and 16 $\mu$M thymidine). Cells were diluted and plated in supplemented DME at 200 and 1,000 viable cells per well (occasionally, at 5,000 viable cells per well also) in 6-well tissue culture plates containing an equal volume of selective medium 2-fold concentrated in selective agent. After 8–10 days, colonies were fixed in methanol-acetic acid, stained with crystal violet, and counted with the aid of a dissecting microscope.

*Determination of cell percentage from colony data*

For each of three experiments, the colony forming efficiencies of viable cells of 4 to 7 168 tumour cell suspensions and the colony forming efficiencies of viable cells of 4 to 9 4T07 tumour cell suspensions were determined in both HAT and thioguanine medium. In all experiments, no colonies were formed from 168 tumour suspensions plated in thioguanine, whereas 4T07 tumour suspensions plated in HAT with an average colony forming efficiency of 0.005%. Most of these colonies were probably host cells, because (a) the colony forming efficiency in HAT medium of 4T07 cells from culture is <0.002%, and (b) the morphology of these colonies and the cells within them differed from 4T07 colonies and cells. Few of these colonies are likely to be HGPRT-positive revertants or fusion products between 4T07 and host cells, because 4T07 tumour suspensions do not form colonies in medium containing HAT plus ouabain. The colony forming efficiency of an individual 168 tumour cell suspension in HAT, and of an individual 4T07 tumour cell suspension in thioguanine medium, was used to analyse the percentage of each cell line existing in each mixture of the two cell suspensions, by using the following formulas:

$xP$ = colony forming efficiency of the mixture in thioguanine.

$y(1-P)$ = colony forming efficiency of the mixture in HAT.

$$y = x \frac{\text{colony forming efficiency in HAT of the line 168 tumour suspension}}{\text{colony forming efficiency in thioguanine of the line 4T07 tumour suspension}}$$

in which:

$x$ is the colony forming efficiency of 4T07 cells in the mixture in thioguanine,

$y$ is the colony forming efficiency of 168 cells in the mixture in HAT, and

$P$ is the proportion of tumour cells which are 4T07.

To analyse mixtures existing in tumour cell suspensions from tumours arising from injection of mixtures of the two cell lines, we determined the mean colony forming efficiences of all the 168 tumours and 4T07 tumours from the same experiment ($n=6$ to 9). These values were used to determine the relationship between $x$ and $y$, as:

$$y = x \frac{\text{mean colony forming efficiency in HAT of all line 168 tumours}}{\text{mean colony forming efficiency in thioguanine of all line 4T07 tumours}}$$

### Results

*Analysis of mixtures of two tumour cell suspensions by DNA content and by colony formation*

We began our analysis of tumour cell content by making mixtures of cell suspensions prepared from homogeneous tumours. In Figure 1 are shown DNA histograms for a normal spleen (Panel A), a typical line 168 tumour (Panel B), a typical line 4T07 tumour (Panel C), a mixture of the two tumour cell suspensions (Panel D), and two tumours resulting from injecting a mixture of 168 and 4T07 cells (Panels E and F). Vertical lines represent windows used to classify cells in each category by DNA content. Spleen cells were used as controls to set the DNA content of diploid cells in each experiment. It is clear that diploid cells in $G_1$ can be clearly distinguished from both tumour cell types, and that the two types of tumour cells can be distinguished from each other fairly well despite some peak overlap. However, the host cells in $G_2$ can not be distinguished from tumour cells and are a significant source of error in estimating the content of a minor tumour population (Figure 1, Panel F).

**Figure 1** DNA histograms from spleen cells and from tumour cell suspensions. Panel A: tumour bearer spleen. Panel B: line 168 tumour suspension. Panel C: line 4T07 tumour suspension. Panel D: 1:1 cell mixture of suspensions from C and D. Panel E: suspension of cells from a tumour which arose from injection of a 1:1 mixture of 168 and 4T07 cells. Panel F: a second tumour arising from injection of the same 1:1 mixture. Vertical lines mark the windows used to classify cells by DNA content: 1, diploid cell window; 2, line 168 cell window; 3, line 4T07 cell window. The vertical axis differs in each panel; in A and F, full scale equals 4000; in B, 1400; in C, 2100; in D, 1600; in E, 3000.

The DNA content per cell of 168 tumour cells was 1.95 times that of spleen cells, and that of 4T07 tumour cells was 2.36 times that of spleen cells. These values are the same as those determined for the $G_0/G_1$ peak of the same tumour cell lines in culture (not shown). In the tumour cell suspensions shown here and in other tumours analysed (a total of 65 tumours), 73 to 90% of the cells analysed were found in the appropriate windows. Other cells were generally scattered throughout the histogram. In some tumours, small peaks could be distinguished at higher DNA levels, which may represent dividing tumour cells, doublets, triplets, and/or cell fusions. Because none of these peaks contained more than 4% of the cells, they were ignored in subsequent analyses.

We also carried out colony forming assays with mixtures of cells from suspensions prepared from homogeneous tumours. In a series of experiments, we prepared cell suspensions from line 168 tumours and 4T07 tumours, mixed these in 9:1, 1:1, and 1:9 cell ratios, and analysed samples of each original suspension and mixture by measuring colony formation in selective media and by DNA content (FACS analysis) to determine the percent of tumour cells which were 4T07. These data are shown in Table I. The FACS data were analysed in two ways: (a) the percent tumour cells *vs.* normal cells in the unmixed suspensions was determined, and these data were used to calculate the expected percent of line 4T07 cells in mixtures (Column 1, Table I); and (b) the mixture itself was analysed by FACS and the 4T07 cell content determined directly (Column II, Table I). These two values agreed well for some mixtures, although in experiment 3 and 4, the percent 4T07 measured directly (II) was considerably lower than that expected (I) in mixtures with high 4T07. These anomalous data were both obtained in a single day. Further analysis of data from these experiments revealed that there was an apparent shift of cells from the diploid peak to the tetraploid (168 tumour cell) peak in mixtures compared to that expected. This shift had the effect of increasing the percent cells classified as 168, thereby decreasing the percent classified as 4T07. The reason for this shift is unknown. Presumably, clumping (dimer formation) of host cells occurred in these samples after mixing the tumour suspensions. In experiment 5, at low 4T07, the measured percent 4T07 was considerably higher than expected. The 168 peak of this sample was quite broad and therefore difficult to analyse. At any rate, data obtained by FACS analyses were responsive to the known dosage of 4T07: mixtures known to contain fewer numbers of 4T07 cells were analysed as such. On an additional 5 pairs of

**Table I** Analysis of mixtures of cell suspensions prepared from homogneous tumours

| | | *Percent of tumour cells which were found to be 4T07:* | | |
|---|---|---|---|---|
| | | *from FACS analysis* | | *from analysis colony formation* |
| *Cell mixture 168:4T07* | *Experiment number*[a] | *I. Expected*[b] | *II. Observed*[c] | *III.*[d] |
| 9:1 | 1. | 5.5 | 10 | 19 |
| | 2. | 6.9 | 14 | 15 |
| | 3. | 6.7 | 4.4 | 22 |
| | 4. | 2.9 | 4.1 | 14 |
| | 5. | 14 | 32 | 8.3 |
| | 6. | 2.5 | 4.1 | 9.1 |
| | Mean ± s.d. | 6 ± 4 | 11 ± 11 | 14 ± 5 |
| 1:1 | 1. | 34 | 34 | 50 |
| | 2. | 40 | 51 | 52 |
| | 3. | 39 | 23 | 38 |
| | 4. | 21 | 5.9 | 39 |
| | 5. | 59 | 64 | 49 |
| | 6. | 19 | 14 | 57 |
| | Mean ± s.d. | 35 ± 15 | 32 ± 22 | 48 ± 8 |
| 1:9 | 1. | 83 | 68 | 90 |
| | 2. | 86 | 88 | 92 |
| | 3. | 85 | 47 | 88 |
| | 4. | 71 | 26 | 82 |
| | 5. | 93 | 84 | 73 |
| | 6. | 68 | 44 | 90 |
| | Mean ± s.d. | 81 ± 10 | 60 ± 24 | 86 ± 7 |

[a]Each experiment used a different pair of tumours; [b]Tumour suspensions before mixing were analysed by FACS for tumour cell *vs.* diploid cell content, and the expected percent of tumour cells which were 4T07 in mixtures was determined from this data; [c]Tumour suspensions after mixing were anlysed by FACS, and the percent of cells in the 4T07 peak was calculated as a percent of the cells in the 4T07 peak plus the 168 peak; [d]Tumour suspensions after mixing were analysed for colony formation in thioguanine medium and in HAT medium, and the percent of tumour cells which were 4T07 was determined as described in **Materials and methods**. The colony forming efficiency used for each cell line in each mixture was that determined for the actual tumour used to make up the mixed suspension.

*Footnote* Tumours arising from injection of line 168 cells or line 4T07 cells were removed, and single cell suspensions were prepared and counted by haemocytometer. No attempt was made to distinguish between tumour cells and other cells such as lymphocytes in haemocytometer counts. Cell suspensions were mixed in ratios as shown, and a sample of each suspension was removed for cloning in selective media. Another sample of each suspension was processed for FACS analysis.

tumours analysed by FACS only, similar relationships were found (mean ± s.d. for method I *vs.* II: at 9:1, 6 ± 3 *vs.* 11 ± 5; at 1:1, 35 ± 10 *vs.* 41 ± 20; at 1:9, 82 ± 6 *vs.* 73 ± 8).

Likewise, colony formation experiments yielded data which were responsive to the known dosage of 4T07. Colony data were in fair agreement with FACS data but tended to yield a higher percent of 4T07. In order to test whether the higher proportion of 4T07 cells was due to an unexpected influence of one subpopulation upon the other in the selective media, we mixed cells from 168 tumour suspensions (200 to 1,000) with 1,000 cells from 4T07 tumour cell suspensions and allowed them to form colonies in HAT. Similarly, we mixed various numbers of cells from 4T07 tumour cell suspensions with 1,000 cells from 168 tumour cell suspensions and allowed them to form colonies in thioguanine. The colony forming efficiences of neither cell line was affected by the presence of the other (Table II). We also added 6 to 50 cells of one type from culture with $10^3$ to $10^5$ cells from tumour suspensions of the second type and plated the mixtures in selective media. These experiments revealed that either minority population could be quantitatively detected even when constituting <0.6% (6 in $10^3$) of the total cell number (Figure 2, Panels A and B). In thioguanine medium, line 4T07 could also be detected when it constituted as little as 0.006% (6 in $10^5$) of the total cells (Figure 2, Panel B), but line 168 colonies could not be distinguished in plates containing $10^5$ line 4T07 tumour suspensions in HAT medium because a lawn of cells was formed in these plates. In this experiment, unlike those of Table II, line 168 cells from culture, which had a high colony forming efficiency in HAT medium, had a slightly lower colony forming efficiency in the presence of 4T07 tumour cell suspensions (Panel A). In contrast, line 168 tumour suspensions had little effect on the colony forming efficiency of line 4T07 cells in thioguanine medium (Panel B). It may be that in some experiments, line 168 is underestimated in the colony formation assay because its actual colony forming efficiency in mixtures is somewhat smaller than it is assumed to be.

From the DNA content assays we were able to determine the percentage of diploid (host) cells in tumours of each type. These data are shown in Table III. Line 4T07 tumour suspensions had a much higher diploid cell content than did line 168 tumours.

### *Analysis of tumours arising from injection of mixtures of two cell lines*

The results with mixtures of homogeneous tumour cell suspensions encouraged us to use FACS analysis and colony formation in selective media to identify these sublines in heterogeneous tumours. We analysed a series of tumours arising from injection of mixtures of 4T07 and 168, at different ratios, by both FACS and colony forming assays. These results are shown in Table IV. There was a strong tendency for tumours to contain a higher percentage of 4T07 than that injected. Only in 2 of the 7 tumours initiated with 19 times as many 168 cells as 4T07 cells were <90% of the tumour cells identified as 4T07 (Table IV). Both techniques detected the same two tumours, one of which contained 39% 4T07 by the colony forming assay, and 50% 4T07 by FACS analysis, whereas the second tumour contained ~85% 4T07 cells by both techniques.

**Figure 2** Colony formation of line 168 cells or line 4T07 cells in selective media in the presence of an excess of the other cell line. Panel A: Line 168 cells from culture plated at the number shown in 60 mm dishes in HAT medium either alone (●) or in the presence of $10^3$ added cells from a 4T07 tumour suspension (○). Panel B: Line 4T07 cells from culture in thioguanine medium either alone (●), with $10^3$ added cells from a 168 tumour suspension (○), or with $10^5$ added cells from a 168 tumour suspension (△). Points, means of 6 replicates; lines, s.e.

**Table II** Colony-forming efficiency of tumour cells when alone and when mixed with cells from another tumour

| *Tumour suspensions* | *Colony-forming efficiency* (%) | |
|---|---|---|
| *168 cells plated in HAT* | *alone* | *with 4T07 added* |
| 1. | 1.7 ± 0.6 (9)[a] | 1.8 ± 0.5 (9) |
| 2. | 6.1 ± 1.6 (9) | 5.9 ± 2.4 (9) |
| 3. | 9.4 ± 2.1 (6) | 7.4 ± 1.6 (6) |
| 4. | 4.7 ± 1.2 (12) | 5.2 ± 1.5 (12) |
| *4T07 cells plated in thioguanine* | *alone* | *with 168 added* |
| 1. | 9.4 ± 2.7 (9) | 8.3 ± 4.3 (9) |
| 2. | 14.4 ± 4.8 (12) | 14.4 ± 2.9 (12) |
| 3. | 7.5 ± 1.7 (12) | 7.1 ± 1.6 (12) |
| 4. | 6.0 ± 1.4 (12) | 7.7 ± 2.4 (12) |

[a]Mean ± s.d. (number of plates).

*Footnote* Four 168 tumour suspensions and four 4T07 tumour suspensions were plated at 200 to 1,000 cells per plate in selective medium, either alone or with the addition of 1,000 cells of tumour suspension from the other tumour cell type.

**Table III** Diploid (host) cell content of tumours determined by FACS analysis

| *Tumour cells injected* | *Diploid cell content (% of total cells)* |
|---|---|
| 168 | 27 ± 7 (18)[a] |
| 4T07 | 54 ± 13 (16) |
| *168:4T07 mixture* | |
| 1:1 | 55 ± 11 (10) |
| 3:1 | 69 ± 13 (7) |
| 9:1 | 59 ± 6 (7) |
| 19:1 | 67 ± 9 (7) |

[a]Mean ± s.d. (number of tumours).

*Footnote* Tumours arising from injection of $3 \times 10^5$ cells, either 168 cells, 4T07 cells or mixtures of the two, were removed, suspended, propidium iodide-stained, and analysed by FACS.

**Table IV** Analysis of tumours arising from mixtures of 168 and 4T07 cells

| *168:4T07 cell ratio injected* | *Fraction of tumours in which the tumour cells were <90% 4T07:* | |
|---|---|---|
| | *by colony formation* | *by FACS analysis* |
| 1:1 | 0/10[a] | 1/10[b] |
| 3:1 | 0/6 | 0/6 |
| 9:1 | 0/7 | 0/7 |
| 19:1 | 2/7[c] | 2/7 |

[a]Number of tumours <90% 4T07/total number of tumours analysed; [b]Tumour cells analysed as 99% 4T07 by colony assay, 83% by FACS; [c]First tumour analysed as 85% 4T07 by colony assay, 84% by FACS. Second tumour analysed as 39% 4T07 by colony assay, 50% by FACS.

*Footnote* Tumours arising from injection of $3 \times 10^5$ total cells were removed, suspended, and assayed by both colony formation and FACS analysis.

Tumours containing ≧90% 4T07 cells could not be further distinguished from each other by FACS because of the broad 4T07 peak overlapping the 168 region of the DNA histogram, because of background noise, and because of host cell $G_2$ overlap with 168 peak (Figure 1, Panel F). The more sensitive colony forming assay, however, allowed a more precise determination of the 168 cell content in these tumours. In tumours arising from a 1:1 injected cell ratio, 6 of 10 were >99.5% 4T07, 3 of 10 were 99%. Similarly, in tumours arising from a 3:1 cell ratio, 3 of 6 were >99.5%, and 3 of 6 were 99%. In tumours arising from 9:1 initial cell ratios, a few more 168 cells could be detected: 2 of 7 tumours were ≧99.5% 4T07, and the remaining tumours were 99, 99, 97, 94, and 93%. In tumours arising from 19:1 cell ratios, 3 of 7 were ≧99.5% 4T07, and the remainder were 97, 92, 85, and 39%.

Although on average we harvested line 168 tumours earlier than line 4T07 tumours (Table V), because of their extremely rapid growth, line 168 tumours tended to be bigger than line 4T07 tumours at the time of harvest (Table V). There was also a larger size range of 168 tumours analysed. Since we used the mean colony forming efficiencies of the unmixed tumours to determine the composition of tumours arising from mixtures, it was important to determine whether the tumour size or day of harvest affected the colony forming efficiency within each group. Multiple regression analysis was used to examine relationships between the colony forming efficiencies of cell suspensions from unmixed tumours in HAT or in thioguanine media, or the percent tumour cells obtained by FACS analysis, with tumour size or day of harvest. No significant correlations were found for either tumour group. Neither were colony forming efficiences significantly correlated with percent tumour cells in either group. In the tumours arising from mixtures, we tested whether the proportion of tumour cells found to be 4T07

**Figure 3** Growth of line 168 tumours, line 4T07 tumours, and tumours arising from mixtures of the two injected at a 19:1 168:4T07 ratio. A total of $3 \times 10^5$ cells were injected s.c. in 0.1 ml Hank's buffer in each group (10 mice per group). Points, means; Lines, s.e. Line 168, ○; 4T07, ●; mixture, △.

was correlated with tumour size or day of harvest within each group. In the 3:1, 9:1, and 19:1 mixtures, these variables were not significantly correlated, but in the 1:1 mixtures the proportions of 4T07 were significantly negatively correlated with day of tumour harvest.

The overgrowth by line 4T07 in mixtures with line 168 would not be expected from examination of the growth rates of the individual tumours (Figure 3). Although line 168 has

**Table V** Size and day of harvest of tumours used in the study

| *Tumour type* | *Number of tumours analysed* | *Tumour size at harvest (mm³)* | *Day of harvest* |
|---|---|---|---|
| 168 | 17 | 1,802±293 (245–4,332)[a] | 28 (21–36)[b] |
| 4T07 | 22 | 738±68 (320–1,800) | 38 (21–43) |
| 168:4T07 mixtures | | | |
| 1:1 | 20 | 825±65 (486–1,666) | 34 (28–47) |
| 3:1 | 6 | 697±98 (405–1,080) | 28 (21–38) |
| 9:1 | 7 | 814±65 (650–1,080) | 35 (28–38) |
| 19:1 | 7 | 497±63 (288–786) | 28 (21–28) |

[a]Mean±s.e. (range); [b]Median (range).

a somewhat longer latency period than does line 4T07, it is a rapidly growing tumour, and would be expected to be the dominant tumour cell by the time of tumour harvest if the cells from mixtures grew independently of one another. Also shown in Figure 3 is the growth curve for tumours arising from injection of mixtures of line 168 plus line 4T07 at a 19:1 cell ratio. The growth of these tumours more closely resembles that of line 4T07. The one tumour of this group which was found to contain 39 to 50 percent 4T07 tumour cells when harvested at day 28, could not be distinguished from the other tumours in its group by growth rate early after injection, but was growing rapidly at the time of harvest.

The diploid host cell content of the tumours arising from mixtures (Table III) was high, as might be expected of tumours which were essentially all 4T07 cells.

### Discussion

We have established a series of mouse mammary tumour cell lines, all derived from the same tumour, which contain drug resistance markers, enabling individual cells to be quantitated in mixtures of cells from tumours or from *in vitro* cultures (B.E. Miller *et al.*, 1983*b*; B.E. Miller *et al.*, 1986). As we describe here, the proportion of each cell line in cell suspensions containing both line 168 and line 4T07 can be determined in mixtures because line 168 (wild type) is able to grow in medium containing HAT but not in medium containing 60 $\mu$M thioguanine, whereas line 4T07 (HGPRT-negative) can grow in thioguanine but not in HAT. In suspensions of cells from tumours, these two types of tumour cells can be distinguished from normal host cells in the colony forming assay because of the much higher colony forming efficiency of the tumour cells under the assay conditions used.

Unfortunately, when we assayed a series of tumours by this method, the total colony forming efficiency (sum of colony forming efficiencies in both media) varied widely from tumour to tumour in tumours arising from either cell line, and in tumours arising from mixtures, so we could not determine directly the actual colony forming efficiency of each cell line within tumours arising from mixtures. The range of colony forming efficiencies for 22 4T07 tumours in thioguanine was 1.2 to 30.0% (mean, 10.7%). The range of colony forming efficiencies for 17 168 tumours in HAT was 1.7 to 20.5% (mean, 8.0%). Therefore, we needed some estimate of the colony forming efficiency of each cell line in mixtures in order to determine the cell proportions from colony numbers. We assumed that the ratio between the colony forming efficiencies of lines 168 and 4T07 remained constant in all tumours containing cell mixtures (i.e., we assumed that whatever factors caused one tumour to have a lower total colony forming efficiency than another tumour, these factors would affect both cell lines proportionally, so that colony forming efficiencies of both cell lines would be lower but remain in the same ratio). We also assumed that this ratio was the same as that found between the mean colony forming efficiencies of the two cell lines in suspensions from unmixed tumours.

The colony formation assay does not require that the cell lines being analysed differ in DNA content. However, because these two cell lines can also be distinguised from each other and from normal host diploid cells in tumour cell suspensions by their DNA content, we were able to test the validity of the above assumptions by assaying the same mixtures by the DNA content assay. As we have shown, there was good agreement between the two assays.

The DNA content assay is somewhat more straightforward than the colony formation assay, because it identifies cells directly. However, errors in cell identification leading to miscalculation of cell proportions occur in this assay, as well. In some tumour cell suspensions, up to 27% of the stained particles counted (average, 21%) did not fall into one of the major peaks. These non-identified particles are most likely doublets, cells in cycle, and debris. They may be more likely to be formed from one cell line than another. Also, there is significant overlap of host $G_2$ and $G_1$ peaks of 168 and 4T07, making precise determination of their proportions impossible. Both of these problems greatly lower the sensitivity of this technique. We estimate that neither cell line can be detected accurately in mixtures unless it is at least 10% of the total tumour cells. If the DNA peaks were farther apart, the sensitivity would be increased but the background noise would still limit sensitivity.

The colony formation assay can detect cells which make up only a very small proportion of the total. In the case of this pair of cell lines, the sensitivity of detection of line 168 in mixtures which are predominantly line 4T07 is somewhat limited, because line 4T07 tumour suspensions form colonies at low frequency (0.005%) in HAT medium. We estimate that line 168 can be detected if it is at least 0.5% of mixtures. However, line 4T07 should be detectable in mixtures which are predominantly 168 with much greater sensitivity, merely by plating more cells. One should be limited only by the need to plate cells at low density to prevent metabolic cooperation. The ability of normal host cells to form colonies in medium containing 60 $\mu$M thioguanine should be extremely small.

Of course, both assays only measure the proportion of the two cell lines in cell suspensions. One or the other cell line may be more fragile under the conditions used to disperse the tumours.

Since both the tumour lines used here have distinctive DNA content greater than that of diploid mouse cells, the DNA content assay also allowed us to quantitate the number of host cells in the tumour samples. The percent of diploid cells in tumours of each type agrees well with the determination of infiltrating host cells by other methods. Our laboratory has found suspensions of 168 tumours to be 12% anti-lymphocyte serum positive (Rios *et al.*, 1983) and 19% Fc receptor positive (Loveless & Heppner, 1983). The sum of these values (31%), agrees very well with the proportion of diploid cells (28%) reported here. We did not determine these values for line 4T07, but line 410.4, the parent line of 4T07, is 38% anti-lymphocyte serum sensitive (Rios *et al.*, 1983), and 24% Fc receptor positive (Loveless & Heppner, 1983). This sum (62%) also agrees very well with the proportion of diploid cells (54%) determined for line 4T07.

The purpose of developing these methods in our laboratory was to be able to quantitate tumour subpopulation interaction within heterogeneous tumours. When mixtures of the two cell lines were injected, the tumour cells which grew out were almost entirely line 4T07, even when we injected a large excess of line 168. Although line 168 tumours have a somewhat longer latency period than line 4T07 tumours, line 168 produces rapidly growing tumours. At the time of tumour harvest, we would not expect either cell line to predominate. It is clear that there is a strong interaction of some kind between tumour cells or between host and tumour which strongly favours the growth of line 4T07 over line 168. We are currently investigating the mechanism of this interaction.

Previously, we reported that line 410 tumours could inhibit the growth of line 168 tumours when the two tumours were growing on opposite sides of the same mouse (B.E. Miller *et al.*, 1980). This interaction, which apparently was due to an immune response to line 410 which, when mounted, could recognise and inhibit the growth of both line 410 and line 168 (Miller *et al.*, 1980), is not the same as the interaction between 168 and line 4T07 described here. Inhibition of line 168 by line 4T07 is very weak when the two cell lines are not in contact, and it appears to take place

*in vitro* under some culture conditions (B.E. Miller & Heppner, manuscript in preparation). The two-site protocol was necessary in investigating interactions between lines 168 and 410 because we were unable to quantitate those lines in mixtures. The existence of 'genetically marked' subpopulation lines overcomes this limitation and allows the study of interactions which depend upon cell contact.

Leith *et al.* (1985) have also described a system of artificial heterogeneous tumours which they have constructed by injecting 1:1 mixtures of two human colon tumour lines, originally isolated from the same tumour, into nude mice. They identified the proportions of the two lines in the tumours which grew out by colony morphology. They found that over time, one cell line overgrew the other, until the tumours appeared to stabilize at a 9:1 mixture of the two cell lines. Other interactions in which the growth of a sister subpopulation is suppressed have been described both *in vitro* (Heppner *et al.*, 1980) and *in vivo* (B.E Miller *et al.*, 1980; Caignard *et al.*, 1985; Newcomb *et al.*, 1978) by mechanisms invoking host immune involvement (B.E. Miller *et al.*, 1980; Caignard *et al.*, 1985; Newcomb *et al.*, 1978) and by mechanisms not involving host immunity (Heppner *et al.*, 1980). Interactions in which the growth of a sister subpopulation is enhanced, have also been described (Caignard *et al.*, 1985; Brodt *et al.*, 1985; Butler *et al.*, 1983; Tofilon *et al.*, 1984), as well as, in some instances, the tendency for two subpopulations to form a particular final ratio (Leith *et al.*, 1985; Jansson & Revesz, 1976).

We feel that the cell lines and the method of analysis described here will be extremely useful in determining the impact of subpopulation interactions on the growth of heterogeneous tumours. In addition, by injecting cell mixtures whose components are differentially sensitive to a given chemotherapeutic agent, we can measure directly the effect of chemotherapy on heterogeneous populations. We have already demonstrated that interactions between cell populations can affect their response to chemotherapy *in vitro* and *in vivo* (B.E. Miller *et al.*, 1981), but we were limited to assay methods which did not allow cells to come in contact. Now that we have available appropriate cell lines and an assay system for identifying these cell lines in mixtures, we can examine such interactions in tumours arising from mixtures of cells.

This work was supported by USPHS Grants CA27419 and CA28366 awarded by the National Cancer Institute. We wish to thank the Ben Kasle Laboratory for Flow Cytometry and the Comprehensive Cancer Center of Metropolitan Detroit, USPHS Grant 22453, for the FACS analysis. We also thank William Illis of the Biostatistics Unit, Michigan Cancer Foundation, for statistical analysis, Donna McInerney for isolation of line 4T07, and Margaret Peterson for manuscript preparation.

### References

BLAZAR, B.A., LAING, C.A., MILLER, F.R. & HEPPNER, G.H. (1980). Activity of lymphoid cells separated from mammary tumors in blastogenesis and Winn assays. *J. Natl Cancer Inst.*, **65**, 405.

BRODT, P., PARHAR, R., SANKAR, P. & LALA, P.K. (1985). Studies on clonal heterogeneity in two spontaneously metastasizing mammary carcinomas of recent origin. *Int. J. Cancer*, **35**, 265.

BUTLER, W.B., TOENNIGES, M.M. & HILLMAN, R.M. (1983). *In vivo* complementation between clones of the human breast cancer cell line MCF-7. *Proc. Am. Assoc. Cancer Res.*, **24**, 35. (*abstract*).

CAIGNARD, A., MARTIN, M.S., MICHEL, M.F. & MARTIN, F. (1985). Interaction between two cellular subpopulations of a rat colonic carcinoma when inoculated to the syngeneic host. *Int. J. Cancer*, **36**, 273.

DEXTER, D.L. & CALABRESI, P. (1982). Intraneoplastic diversity. *Biochem. Biophys. Acta.*, **695**, 97.

DEXTER, D.L., KOWALSKI, H.M., BLAZAR, B.A., FLIGIEL, Z., VOGEL, R. & HEPPNER, G.H. (1978). Heterogeneity of tumor cells from a single mouse mammary tumor. *Cancer Res.*, **38**, 3174.

FIDLER, I.K. & HART, I.R. (1982). Biological diversity in metastatic neoplasms: Origins and implications. *Science*, **217**, 998.

HEPPNER, G.H. (1984). Tumor heterogeneity. *Cancer Res.*, **44**, 2259.

HEPPNER, G.H., DEXTER, D.L., DENUCCI, T., MILLER, F.R. & CALABRESI, P. (1978). Heterogeneity in drug sensitivity among tumor cell subpopulations of a single mouse mammary tumor. *Cancer Res.*, **38**, 3758.

HEPPNER, G.H. & MILLER, B.E. (1983). Tumor heterogeneity: Biological implications and therapeutic consequences. *Cancer Metastasis Rev.*, **2**, 5.

HEPPNER, G.H., MILLER, B., COOPER, D.N. & MILLER, F.R. (1980). Growth interactions between mammary tumor cells. In *Cell Biology of Breast Cancer*, McGrath, C. *et al.*, (eds) p. 166. Academic Press: New York.

JANSSON, B. & REVESZ, L. (1976). A deductive approach to the analysis of the growth of ascites tumor cell populations. *Meth. Cancer Res.*, **13**, 227.

LEITH, J.T., FAULKNER, L.E., BLIVEN, S.F., LEE, E.S., GLICKSMAN, A.S. & DEXTER, D.L. (1985). Dissaggregation studies of xenograft solid tumors grown from pure or admixed clonal subpopulations from a heterogeneous human colon adenocarcinoma. *Invasion Metastasis*, **5**, 317.

LOVELESS, S.E. & HEPPNER, G.H. (1983). Tumor-associated macrophages of mouse mammary tumors. I. Differential cytotoxicity of macrophages from metastatic and non-metastatic tumors. *J. Immunol.*, **131**, 2074.

MILLER, B.E., MCINERNEY, D., JACKSON, D. & MILLER, F.R. (1986). Metabolic cooperation between mouse mammary tumor subpopulations in three-dimensional collagen gel cultures. *Cancer Res.*, **46**, 89.

MILLER, B.E., MILLER, F.R. & HEPPNER, G.H. (1981). Tumor heterogeneity and drug sensitivity: Interactions between tumor subpopulations affecting their sensitivity to the antineoplastic agents cyclophosphamide and methotrexate. *Cancer Res.*, **41**, 4378.

MILLER, B.E., MILLER, F.R. & HEPPNER, G.H. (1983*a*). Development of a drug-sensitivity assay for heterogeneous tumors based on growth in 3-dimensional collagen gels. In *Rational Basis for Chemotherapy*, Chabner, B.A., (ed) p. 107. Alan R. Liss, Inc.: New York.

MILLER, B.E., MILLER, F.R. & HEPPNER, G.H. (1984). Assessing tumor drug sensitivity by a new *in vitro* assay which preserves tumor heterogeneity and subpopulation interactions. *J. Cell. Physiol. Suppl.*, **3**, 105.

MILLER, B.E., MILLER, F.R. & HEPPNER, G.H. (1985). Factors affecting growth and drug sensitivity of mouse mammary tumor lines in collagen gel culture. *Cancer Res.*, **45**, 4200.

MILLER, B.E., MILLER, F.R., LEITH, J. & HEPPNER, G.H. (1980). Growth interaction *in vivo* between tumor subpopulations derived from a single mouse mammary tumor. *Cancer Res.*, **40**, 3977.

MILLER, B.E., ROI, L.D., HOWARD, L.M. & MILLER, F.R. (1983*b*). Quantitative selectivity of contact-mediated intercellular communication in a metastatic mouse mammary tumor line. *Cancer Res.*, **43**, 4102.

MILLER, F.R. (1983). Tumor subpopulation interactions in metastasis. *Invasion Metastasis*, **3**, 234.

MILLER, F.R., MEDINA, D. & HEPPNER, G.H. (1981). Preferential growth of mammary tumors in intact mammary fatpads. *Cancer Res.*, **41**, 3863.

MILLER, F.R., MILLER, B.E. & HEPPNER, G.H. (1983). Characterization of metastatic heterogeneity among subpopulations of a single mouse mammary tumor: Heterogeneity in phenotypic stability. *Invasion Metastasis*, **3**, 22.

NEWCOMB, E.W., SILVERSTEIN, S.C. & SILAGI, S. (1978). Malignant mouse melanoma cells do not form tumors when mixed with cells of a non-malignant subclone: Relationships between plasminogen activator expression by the tumor cells and the host's immune response. *J. Cell Physiol.*, **95**, 169.

POSTE, G. & GREIG, R. (1982). On the genesis and regulation of cellular heterogeneity in malignant tumors. *Invasion Metastasis*, **2**, 137.

RIOS, A.M., MILLER, F.R. & HEPPNER, G.H. (1983). Characterization of tumor-associated lymphocytes in a series of mouse mammary tumor lines with differing biological properties. *Cancer Immunol. Immunother.*, **15**, 87.

TOFILON, P.J., BUCKLEY, N. & DEEN, D.F. (1984). Effect of cell-cell interactions on drug sensitivity and growth of drug-sensitive and -resistant tumor cells in spheroids. *Science*, **226**, 862.